# RANBP1 promotes immune evasion in triple-negative breast cancer by suppressing T cell infiltration via the miR-769-5p/PRUNE2 axis

**DOI:** 10.1007/s12672-025-03872-7

**Published:** 2025-11-04

**Authors:** Pengxia Song, Xianglin Liu, Huiping Qiu, Yanhui Cao, Jie Liu, Xin Zheng, Shuihong Yao, Meng Cao

**Affiliations:** 1 Medical School, Quzhou College of Technology, No.18, Jiangyuan Road, Quzhou, 324000 Zhejiang PR China; 2 Department of General Surgery, Nanjing Drum Tower Hospital, the Affiliated Hospital of Nanjing University Medical School, Nanjing, 210008 Jiangsu PR China

**Keywords:** Breast cancer (BC), RANBP1, miR-769-5p, PRUNE2, Motility, Tumorigenesis

## Abstract

**Background:**

T cell dysfunction in the tumor microenvironment (TME) is a major obstacle to effective immunotherapy in triple-negative breast cancer (TNBC). The molecular mechanisms underlying T cell exclusion remain poorly understood.

**Purpose:**

This study identifies RANBP1 as an oncogenic factor in TNBC and investigates its role in modulating T cell infiltration and tumor progression.

**Methods:**

Single-cell and bulk RNA sequencing were used to assess immune cell infiltration associated with RANBP1 expression. RANBP1 protein levels were evaluated in 87 TNBC tumor and adjacent normal tissues by immunohistochemistry. Kaplan–Meier analysis was used to assess overall survival. In vitro and in vivo assays were performed to explore the RANBP1/miR-769-5p/PRUNE2 pathway.

**Results:**

scRNA-seq identified 10 cell types in the TNBC TME. High RANBP1 expression correlated with increased tumor cells, B cells, macrophages, and epithelial cells, and reduced T cells. Cell–cell communication was enhanced in the high-RANBP1 group. TCGA and GSE65194 data confirmed decreased CD4⁺ T cells and Tregs in high-RANBP1 tumors. RANBP1 was significantly upregulated in TNBC and associated with poor prognosis. Functional studies showed that RANBP1 promotes TNBC cell proliferation and migration. Mechanistically, RANBP1 upregulates oncogenic miR-769-5p, which suppresses PRUNE2, a tumor suppressor that normally inhibits TNBC progression.

**Conclusions:**

RANBP1 shapes an immunosuppressive microenvironment in TNBC by reducing T cell infiltration through the miR-769-5p/PRUNE2 axis. These findings reveal a novel immune escape mechanism and suggest that targeting RANBP1 may enhance immunotherapy efficacy in TNBC.

**Supplementary Information:**

The online version contains supplementary material available at 10.1007/s12672-025-03872-7.

## Background

Breast cancer (BC) is recognized as the leading cause of cancer-associated mortality among women [1]. Based on the PAM50 gene-expression classification, BC comprises five distinct molecular subtypes: normal-like, HER-2 enriched, Luminal A, Luminal B, and basal-like or TNBC [2, 3]. TNBC typically correlates with unfavorable clinical outcomes, characterized by aggressive tumor growth, rapid metastatic dissemination, high heterogeneity, and limited therapeutic options [4, 5]. Nonetheless, the precise molecular mechanisms underlying the invasive and metastatic potential of TNBC are not yet fully elucidated.

Non-coding RNA (ncRNA) have emerged as critical regulators in cancer biology [6]. MicroRNAs (miRNAs), a subgroup of ncRNAs typically comprising 18–22 nucleotides, have been reported to significantly modulate BC initiation, progression, and metastasis [6, 7]. Previous studies indicated miRNAs possess oncogenic capabilities in TNBC, notably by enhancing tumor cell invasion and proliferation via interactions with the PTEN signaling cascade [8]. Moreover, TNBC cell proliferation was promoted through miR-3143-mediated stimulation of the PI3K/AKT cascade [9]. On the other hand, miR-143-3p acts as a suppressor of tumor growth via inhibition of PI3K/Akt pathway signaling [10]. Additionally, TNBC progression is influenced by miR-210-3p, which regulates the Wnt/β-catenin pathway by targeting NFIX [11]. However, detailed insights into the upstream regulatory pathways controlling these miRNAs in TNBC remain limited.

Located on chromosome 22, the Hpall Tiny Fragments Locus 9 A (HTF9A) gene encodes Ran-binding protein 1 (RANBP1) [12]. This protein is part of the small GTPase group, itself a member of the broader RAS superfamily [12, 13]. RANBP1 plays multiple essential roles within cellular biology, including facilitating miRNA nuclear-to-cytoplasmic transport, aiding the maturation process of precursor miRNAs, controlling cell cycle checkpoints, and ensuring proper mitotic progression [14]. Increased expression of RANBP1 has been observed in BC tissues, where it serves as an independent prognostic marker associated with poor outcomes [15]. Our earlier research demonstrated that RANBP1 is involved in colorectal cancer progression; however, the precise functions and significance of RANBP1 in TNBC progression and metastatic events have not yet been fully explored.

RANBP1 affects the tumor immune microenvironment and was found to be over-expressed in TNBC patient samples during the current work. The effect of RANBP1 on miR-769-5p and the RANBP1/miR-769-5p/prune homolog 2 (PRUNE2) axis with implications for TNBC progression and metastasis were investigated. The aim was to evaluate the therapeutic potential of targeting the RANBP1/miR-769-5p/PRUNE2 signaling pathway for TNBC treatment.

## Methods

### Data collection and processing

The datasets utilized in this research, including scRNA-seq data (GSE176078) and bulk RNA-seq data (GSE65194), were downloaded from the Gene Expression Omnibus (GEO). Specifically, dataset GSE176078 comprises single-cell RNA-seq profiles derived from ten TNBC tumor specimens, whereas dataset GSE65194 consists of bulk RNA-seq information from 69 TNBC samples. TCGA was also used to gather clinical annotations and gene expression data related to breast cancer (BRCA). As a result of clinical data filtering, 88 instances of TNBC were identified within the TCGA-BRCA cohort. Based on the median RANBP1 expression values, samples from each dataset (GSE176078, TCGA-TNBC, and GSE65194) were then categorized as having high or low RANBP1 expression.

### ScRNA-seq analysis

Seurat was used to analyze the scRNA-seq data. At the outset, cells with mitochondrial gene expression levels above 20%, genes found in less than three cells, or fewer than 300 genes were screened out for rigorous quality control. After this, the “NormalizeData” and “ScaleData” functions were used to normalize and scale the gene expression matrices. Statistical evaluation led to the retention of the first 30 principal components after performing principal component analysis (PCA). By utilizing the “RunHarmony” technique, batch effects were mitigated across distinct single-cell datasets. Uniform manifold approximation and projection (UMAP) was used to reduce the dimensionality. Annotations were made to cell populations by comparing them to the CellMarker 2.0 database. T cells were isolated and then subgrouped for in-depth labeling. We used the CellChat R program to examine intercellular communication inside and between the groups with high and low RANBP1 expression, and we visualized the differences in cell composition between the two groups.

### Bulk RNA-seq analysis

We used the R package “IOBR.” and eight separate bioinformatics tools to evaluate ICI in the TCGA-TNBC and GSE65194 cohorts: CIBERSORT, EPIC, MCPcounter, xCELL, ESTIMATE, TIMER, quanTIseq, and IPS. Afterwards, the groups with high and low RANBP1 expression levels were compared and their immune infiltration characteristics were analyzed.

### Clinical specimen collection

Data came from 87 patients with TNBC who had breast surgery at the Breast Surgery Center of Nanjing Drum Tower Hospital between 2012 and 2018. The samples included both tumor and surrounding non-malignant tissue. Up until December 2023, we continued to follow up on survivors. The fact that no patients had received radiation or chemotherapy prior to surgery is crucial. You may find a thorough summary of the cohort’s clinicopathological features in Table 1. The Ethics Committee of Nanjing Drum Tower Hospital evaluated and approved this research protocol (approval no.: 2023-354-01). Prior to sample collection, all subjects were asked to provide written informed permission.

**Table 1:** Clinical characteristics of triple-negative breast cancer in RANBP1-low group and RANBP1-high group

	Group	RANBP1-low group (*n*=56)	RANBP1-high group (*n*=31)	*P* value
Average ages (year)		48.7	59.2	
Vital status	Alive	50	18	0.0021
	Dead	6	13	
5 years survival rate		53/56(94.6%)	19/31(61.3%)	0.0002
Stage	I-II	**54**	**18**	<0.0001
	I	21	6	
	II	33	12	
	III-IV	**2**	**13**	
	III	2	12	
	IV	0	1	
T stage	T1	**25**	**9**	0.1752
	T2-T3	**31**	**22**	
	T2	26	20	
	T3	5	6	
N stage	N0	**43**	**12**	0.0009
	N1-N3	**13**	**19**	
	N1	11	8	
	N2	2	4	
	N3	0	7	
M stage	M0	**56**	**30**	0.3636
	M1	**0**	**1**	

### Cell lines and culture conditions

Human BC cell lines BT-549 and MDA-MB-231, along with human embryonic kidney HEK 293T cells, were obtained from Shanghai Institute of Cell Biology’s Cell Bank. Cell cultivation involved growing HEK 293T and MDA-MB-231 cells in DMEM medium (Beyotime, China), supplemented with 10% fetal bovine serum (FBS), whereas BT-549 cells were maintained in RPMI-1640 medium with the same concentration of FBS. Regular monitoring using the Myco-Zero Detection Kit (Beyotime, China) verified the absence of mycoplasma in all cell lines.

###  Reagents, plasmids, transfection and lentivirus infection

Negative control oligonucleotides, as well as mimics and inhibitors for miR-769-5p utilized in this study, were procured from Guangzhou RiboBio Co., Ltd. The lentiviral constructs employed—including shRNAs targeting PRUNE2 and RANBP1, corresponding negative controls (sh-NC), and expression vectors for PRUNE2 and RANBP1 overexpression alongside their empty vectors (OE-Vector)—were developed by Nanjing Tsingke Biotechnology. Lentiviral particles were produced by transiently transfecting HEK 293T cells for 48 h using Lipofectamine 3000 reagent (Thermo Fisher Scientific, China) with lentivirus-packaging plasmids (psPAX2 and pMD2.G) and the relevant target vectors. Subsequently, the transfection effectiveness was confirmed at both the protein and RNA levels via Western blot (WB) and RT-qPCR analyses, respectively.

### qPCR analysis

Total RNA was initially isolated utilizing TRIzol reagent (Takara, China). Subsequently, cDNA synthesis was performed using either Takara’s RT reagent kit or Vazyme Biotech’s HiScript III 1st Strand cDNA Synthesis Kit, which includes a genomic DNA removal step. Applied Biosystems’ (FB, CA, USA) ABI PCR apparatus and Vazyme Biotech Co., Ltd.‘s (Nanjing, China) SYBR Green Master Mix were used to perform real-time quantitative PCR (RT-qPCR). Utilizing miRNA-specific reverse transcription primers and the qRT-PCR Starter Kit acquired from Ribobio (China), the miR-769-5p expression levels were measured. Table 2 contains all of the primer sequences needed for qPCR.

**Table 2:** Primer used during qRT-PCR

Sequence type	Sequence (5’–3’)
RANBP1	
Forward primer	GCTCGGGACGAGGATCAC
Reversed primer	CTCTCTCTTCGATCTCTTTCCTG
PRUNE2	
Forward primer	GGGTCTTCTGGGATTATGG
Reversed primer	CTGGGCTAACAAGGTCTAC
U6	
Forward primer	CTCGCTTCGGCAGCACA
Reversed primer	AACGCTTCACGAATTTGCGT
Actin	
Forward primer	CTATCACCTCCCCTGTGTG
Reversed primer	TCCCTTGCCCTCCTAAA
miR-769-5p mimic	UGAGACCUCUGGGUUCUGAGCU
anti-miR-769-5p	AGCUCAGAACCCAGAGGUCUCA
miR-NC	UCACAACCUCCUAGAAAGAGUAGA
anti-miR-NC	UCU ACUCUUUCUAGGAGGUUGUGA

### Cellular activities assays

Cell proliferation assays involved seeding cells into 96-well plates at 3,000 cells per well. Daily evaluations from day one through four utilized CCK-8 reagent (Beyotime, China). Specifically, 10 µL CCK-8 reagent was dispensed into each well, plates were incubated for 1 h at 37 °C. Additionally, colony formation capability was assessed by plating 1,000 cells per well in 6-well plates, incubating until visible colonies developed.

Cell migration potential was evaluated through Transwell assays. Specifically, cells (2 × 10^4^) were seeded in serum-free DMEM or RPMI-1640 medium into the upper chambers, while the lower chambers were filled with medium supplemented with 20% FBS as a chemoattractant. Following 72-hour incubation, cells migrating through the membrane were fixed, stained with crystal violet, and enumerated under microscopy.

To find out how invasive it could be, the same experimental settings as stated for migration experiments were utilized, using Transwell inserts (8 μm pores; Corning, USA) covered with Matrigel (BD Biosciences via Beyotime, China).

### luciferase reporter assay and transfection

We generated luciferase reporter vectors comprising wild-type or mutant variants of the PRUNE2 3’UTR in order to evaluate the direct interaction. Subsequently, HEK 293T cells underwent co-transfection using these plasmids combined with either miR-769-5p mimics or matched negative control miRNAs (miR-NC). After 48 h of transfection, luciferase activity was measured employing the Dual-Luciferase Reporter Assay Kit (Promega, Madison, WI, USA).

### WB

Following cellular lysis on ice using RIPA buffer, proteins were resolved by 10% SDS-PAGE electrophoretic analysis. Subsequently, the separated proteins were electro-transferred onto PVDF membranes (0.45 μm, Millipore). The membranes underwent initial blocking with 5% skim milk, then incubation at approximately 4 °C overnight (around 10 h) with primary antibodies directed against Tubulin, Ki67, PRUNE2, and RANBP1 (Proteintech, China; dilution 1:1000). After multiple washes, membranes were incubated for 1 h with secondary antibodies. The protein bands were then detected using the BeyoECL Plus chemiluminescence detection reagent (Beyotime, China). Due to differences in chemiluminescent exposure bands for different target proteins, some membranes were cropped to clearly present the relevant protein signals. Uncropped original images with visible membrane edges for all replicates are provided in the Supplementary Information.

### Nude mouse tumor xenograft models

BALB/c female nude mice (4–5 weeks old) were purchased from the Model Animal Research Center of Nanjing University. MDA-MB-231 cells stably infected with lentiviruses expressing shRNA targeting RANBP1 or a corresponding negative control vector were subcutaneously implanted into the dorsal flank area of each mouse. Tumor dimensions were recorded using calipers every two days. The mice were sacrificed 28 days post-implantation, followed by the excision of tumors to assess their volume and weight.

### Immunohistochemistry (IHC) analysis

Tumors were fixed, embedded in paraffin, and subsequently sliced into 5 μm-thick sections. Tissue sections underwent overnight incubation (approximately 18 h) at 4 °C with primary antibodies targeting Ki67, PRUNE2, and RANBP1. After phosphate-buffered saline (PBS) washes, the sections were further incubated for two hours with appropriate secondary antibodies. Immunohistochemically labeled slides were examined microscopically at 100× magnification.

### Statistical methods

Data were analyzed utilizing GraphPad Prism (version 10.0) and X-Tiles [16]. Data were presented as mean values ± standard deviations (SD). Comparisons were conducted via Student’s t-test, considering p-values below 0.05 as statistically meaningful. KM analyses were applied to construct survival curves. Levels of statistical significance were represented as *P < 0.05, **P < 0.01, and ***P < 0.001.

## Results

### scRNA-seq analysis

**Fig. 1:**
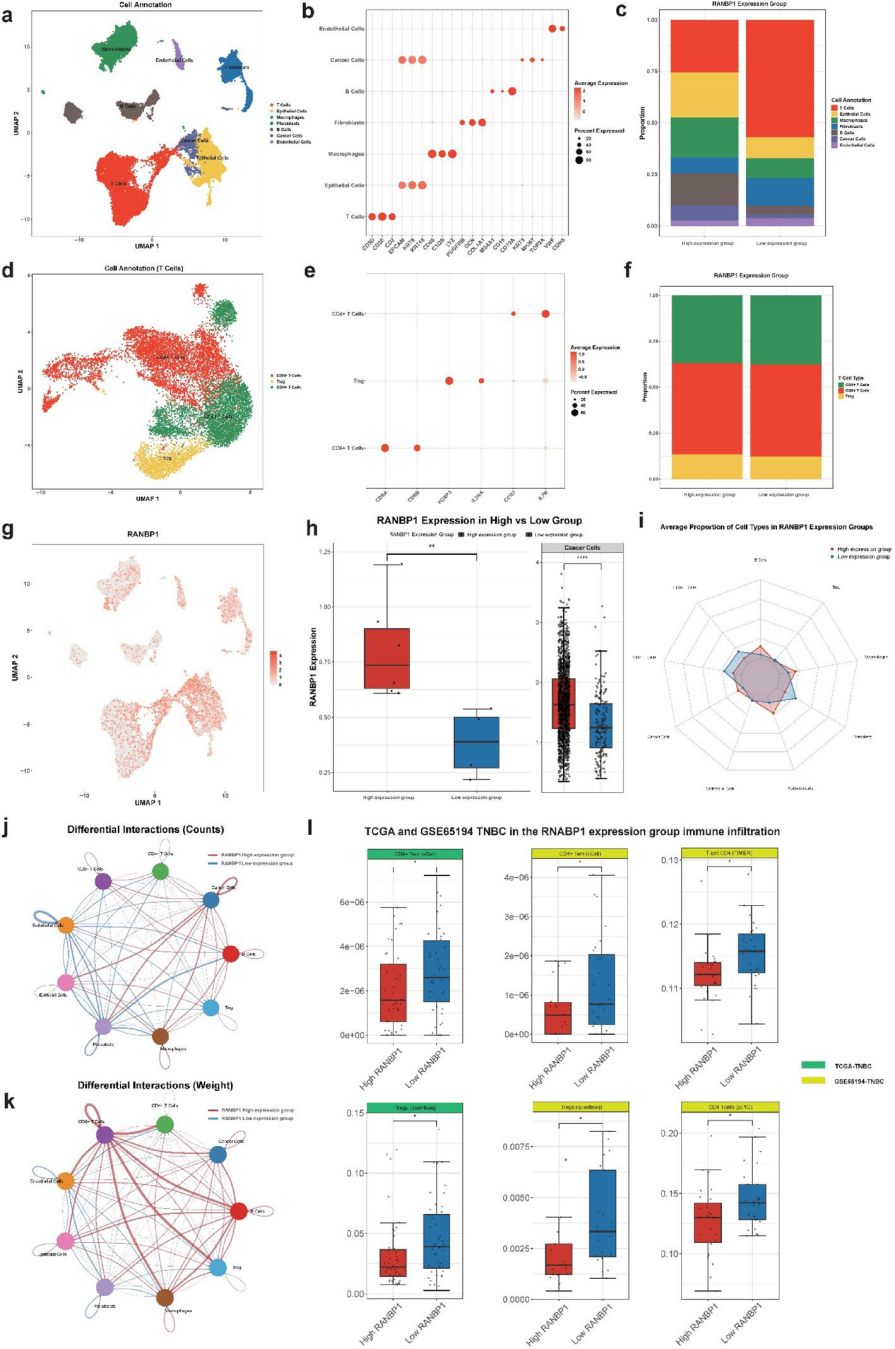
Single-cell transcriptomic and immune infiltration analysis reveals the association between RANBP1 expression and the tumor immune microenvironment in TNBC. **a** UMAP plot showing clustering and annotation of major cell types in the TNBC TME, including T cells, B cells, ECs, cancer cells, endothelial cells, fibroblasts, and macrophages. **b** Dot plot showing the expression levels and proportion of cells expressing canonical marker genes used for annotation of major cell types. **c** Stacked bar plot displaying the proportion of major cell types in the high vs. low RANBP1 expression groups. **d** UMAP visualization of T cell subtypes, including CD4⁺ T cells, CD8⁺ T cells, and regulatory T cells (Tregs). **e** Dot plot showing expression of canonical marker genes used for annotation of T cell subtypes. **f** Proportional distribution of T cell subtypes between high and low RANBP1 expression groups. **g** UMAP feature plot showing the expression of RANBP1 across single cells. **h** Box plot comparing RANBP1 expression between high and low expression groups (*p* < 0.01). **i** Radar plot summarizing the average proportion of immune cell types in the two RANBP1 expression groups. **j** Differential intercellular communication network (based on interaction counts) between RANBP1 high and low expression groups. **k** Differential communication network based on interaction strength (weights) between the two groups. **l** Box plots showing immune infiltration levels of CD8⁺ T cells, CD4⁺ T cells, Tregs, and other immune subsets in RANBP1 high vs. low expression groups across TCGA and GSE65194 TNBC datasets

By integrating data normalization, scaling, and PCA, a total of 21 distinct cell clusters were generated. These clusters were subsequently annotated into seven major cell populations according to known cell-type-specific markers (Figs. 1a, b). A comparative assessment revealed compositional variations among these cell populations between groups defined by high and low RANBP1 expression. Specifically, higher frequencies of tumor cells, ECs, B cells, and macrophages were observed in the group exhibiting elevated RANBP1 expression, while T cells predominated in the low expression cohort (Fig. 1c). Furthermore, T cell subsets were subdivided into CD4 + T cells, CD8 + T cells, and Tregs, based on additional marker genes (Figs. 1d, e). T cell ratio analysis showed that CD4 + T cells and Treg cells were decreased in the RANBP1 high expression group compared with the low expression group (Fig. 1f, S5a). Our immunofluorescence results showed that CD4 + T cells and Treg cells exhibited the same trend (Fig. S6a, b). Moreover, analyses of expression patterns indicated widespread expression of RANBP1 across various cell populations, with significantly elevated overall levels observed in the group classified as high-expression (*p* < 0.01) (Figs. 1g, h). Importantly, tumor cells specifically showed notably increased RANBP1 expression in the high-expression subset (*p* < 0.0001) (Fig. 1h). Further analysis via radar plots quantified these compositional changes, highlighting increased proportions of B cells and macrophages accompanied by reduced frequencies of CD4 + T cells, CD8 + T cells, and Tregs in the high RANBP1-expression group, suggesting a regulatory role for RANBP1 in immune cell composition within the TME (Fig. 1i). Additionally, analyses of intercellular communication demonstrated substantial differences in both interaction frequency and intensity between the high and low RANBP1 expression groups (Figs. 1j, k).

### Analysis of immune cell infiltration

To verify Immune cell infiltration, eight widely recognized computational approaches were applied to TNBC datasets obtained from TCGA and GSE65194, comparing the high and low RANBP1 expression groups. Immune infiltration profiles were illustrated through heatmaps and boxplots (Supplementary Figs. 1–3). Corroborating the single-cell RNA sequencing findings, the high-expression RANBP1 group demonstrated notably reduced infiltration of immune cell subsets, particularly CD4 + T cells and Tregs (Fig. 1l).

### High RANBP1 expression was associated with TNBC progression

**Fig. 2:**
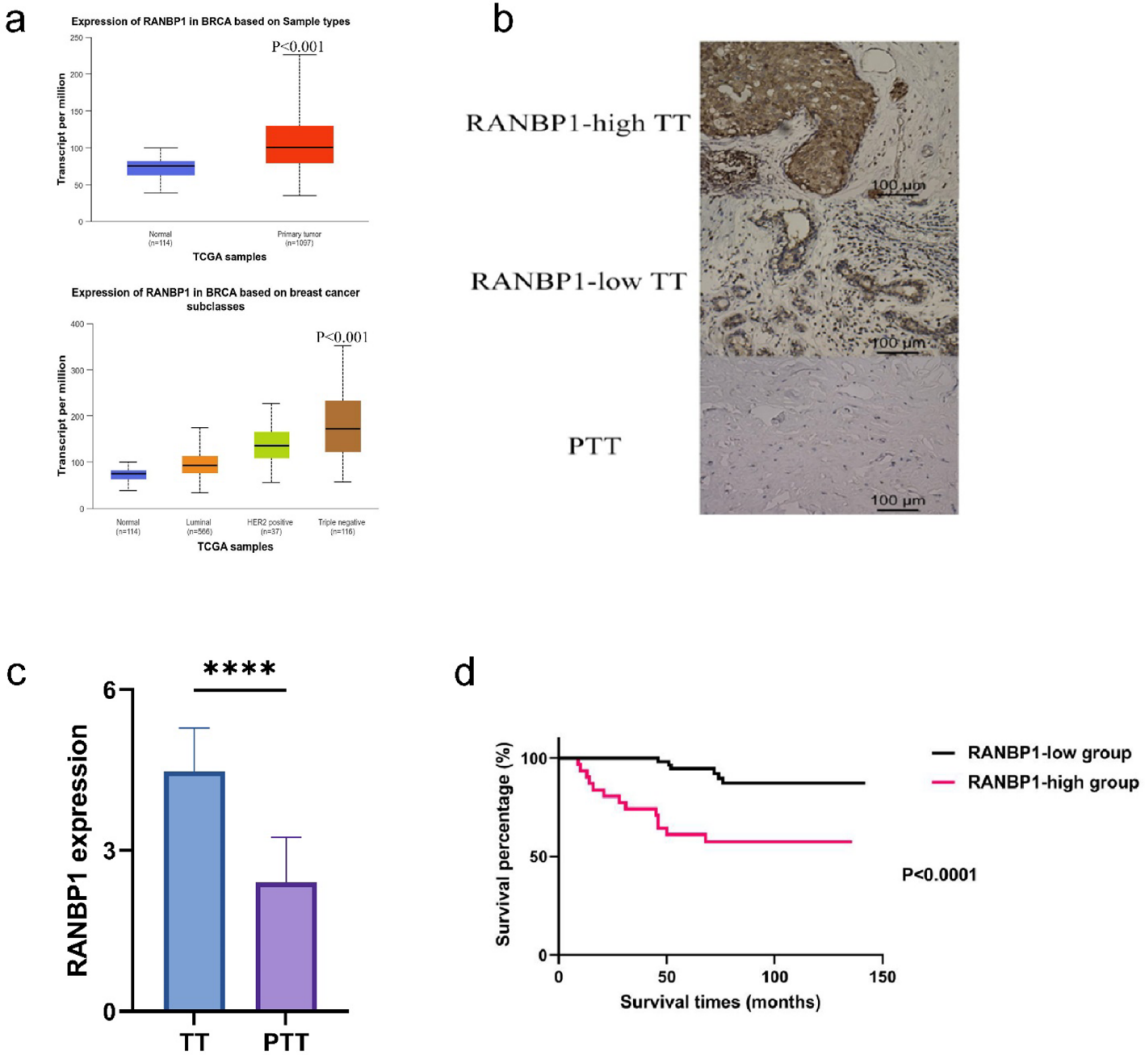
Elevated expression of RANBP1 in TNBC. RANBP1 is up-regulated in breast tissues from the TCGA (**a**); IHC images of RANBP1 in cancerous and adjacent non-cancerous tissues from 87 patients included in the study (scale bar of 100 μm at 200x magnification) (**b**); The expression levels of RANBP1 between the cancerous and adjacent non-cancerous tissues (**c**); The overall KM survival curves between RANBP1-low group and RANBP1-high group (**d**). *TT* Tumoral tissues or cancerous tissues

RANBP1 was over-expressed in tumor samples from TCGA-BRCA database (Fig. 2a). IHC staining of 87 pairs of cancer tissues and paraneoplastic tissues showed RANBP1 to be significantly up-regulated in TNBC tissues (Fig. 2b, c).

IHC analysis was performed on paired tumor and adjacent normal tissue specimens from 87 TNBC patients, stratified into low RANBP1 (*n* = 56) and high RANBP1 (*n* = 31) expression cohorts based on the differential expression threshold (Table 1). There were significant differences in vital status, 5-year survival, tumor stage, and N stage between these two groups; however, T and M stages showed no significant differences (Table 1). KM curves revealed markedly poorer prognosis in patients with high RANBP1 expression (Fig. 2d), suggesting that increased RANBP1 levels promote disease progression in TNBC. Furthermore, we used an independent external validation set (GSE58812) to validate the prognostic correlation by stratifying RANBP1 expression into high and low expression groups. The results also showed that patients with high RANBP1 expression also had a significantly worse prognosis (Fig. S5b).

### RANBP1 promoted cell motility and proliferation in vitro

**Fig. 3:**
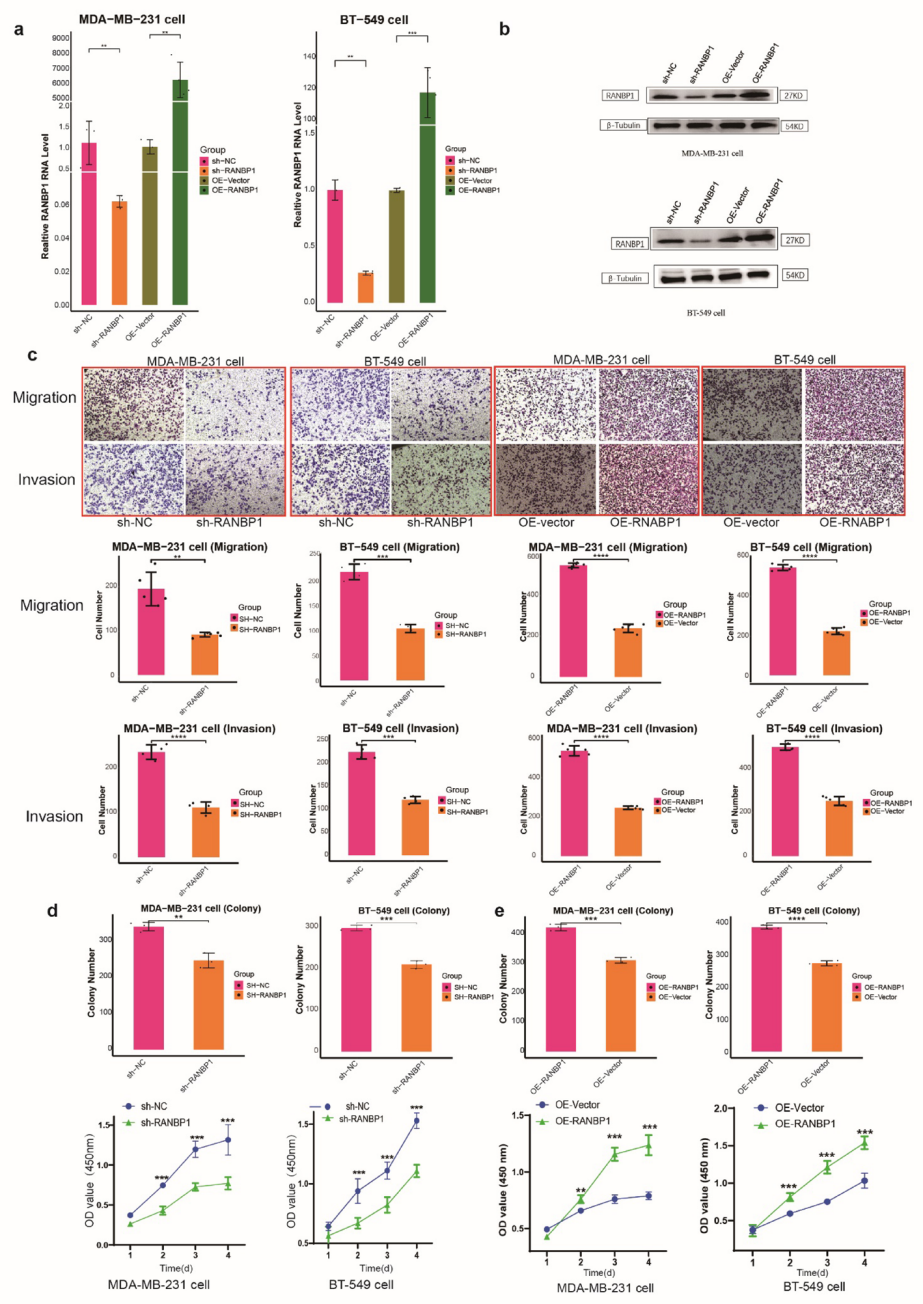
Effect of RANBP1 on the proliferation and migration of the BC cells. Expression of RANBP1 silencing or over-expression was detected in MDA-MB-231 and BT-549 cells by qRT-PCR (**a**) and by WB (**b**). Cell migration and invasion of RANBP1 silencing (**c**) or over-expression (**d**) determined by transwell assay in MDA-MB-231 cells and BT-549 cells. CCK-8 and Colony formation assay was applied to measure the MDA-MB-231cell and BT-549 cells proliferation in RANBP1 silencing (**e**) or over-expression (**f**). *P < 0.05, **P < 0.01 and ***P < 0.001

**Fig. 4:**
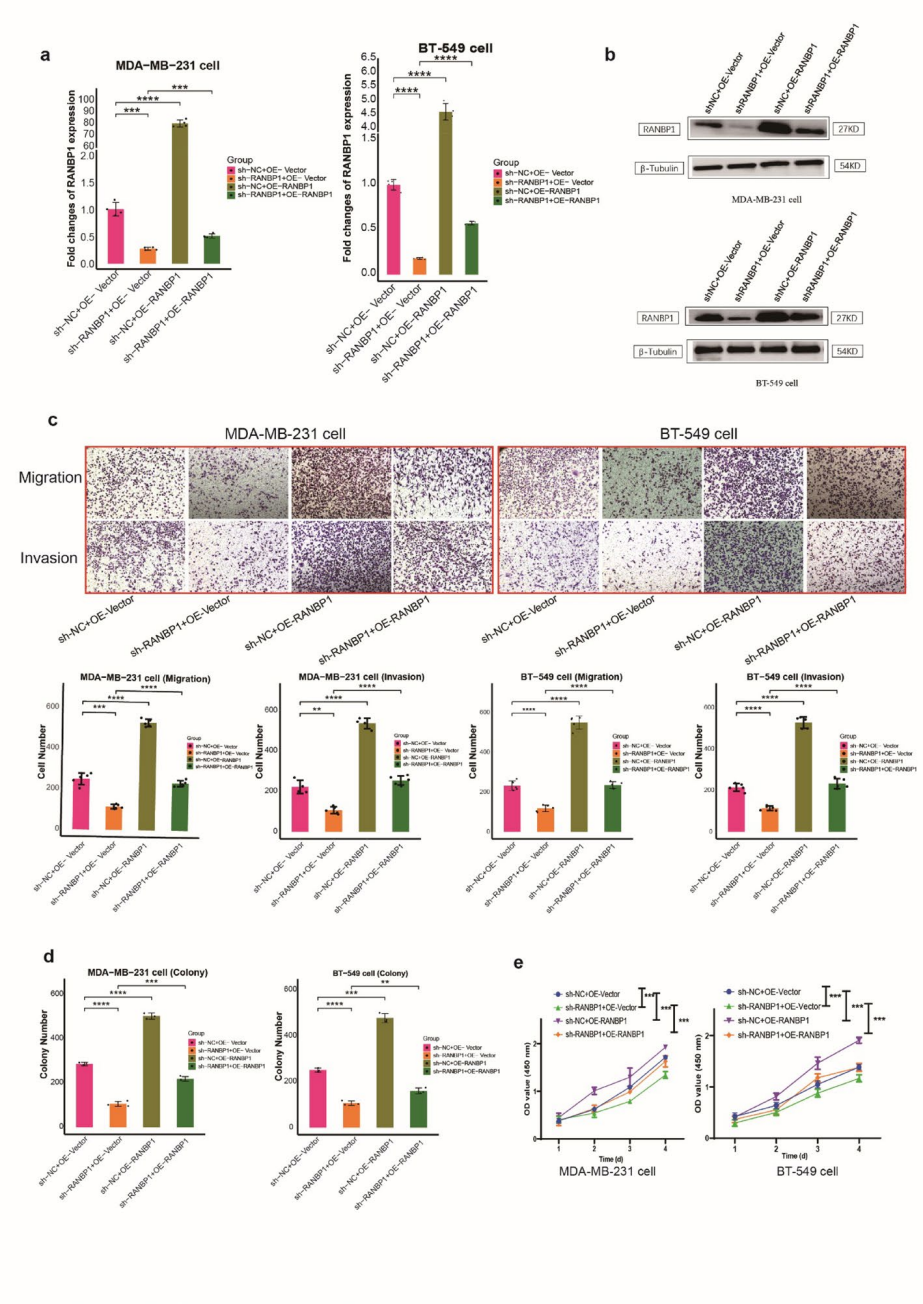
Cells were grouped with sh-NC + OE-vector, sh-RANBP1 + OE-vector, sh-NC + OE-RANBP1 and sh-RANBP1 + OE-RANBP1. (a) qRT-PCR, (b) WB, (c) transwell assay, (d) colony formation, and (e) CCK8 were detected, respectively

Over-expression and knock out of RANBP1 was verified in TNBC cell lines, MDA-MB-231 and BT-549, by qRT-PCR and WB (Fig. 3a, b). RANBP1 knockdown led to decreased rates of cell migration and invasion and RANBP1 over-expression had the opposite effect (Fig. 3c). Similar results were seen for proliferation assays with RANBP1 overexpression increasing rates and RANBP1 knockdown decreasing rates (Fig. 3d, e, S6). Rescue experiments in which the RANBP1 knockdown phenotype was changed by addition of the RANBP1 over-expressing plasmid showed that replenishment of RANBP1 restored levels of cell migration and invasion (Fig. 4a, b, c). Proliferation and clone formation were similarly enhanced by co-transfection of OE-RANBP1 + sh-RANBP1 compared with the sh-RANBP1group (Fig. 4d, e, S6). The results imply a role for RANBP1 in promoting BC cell growth, migration and invasion.

### RANBP1 stimulated miR-769-5p in TNBC

**Fig. 5:**
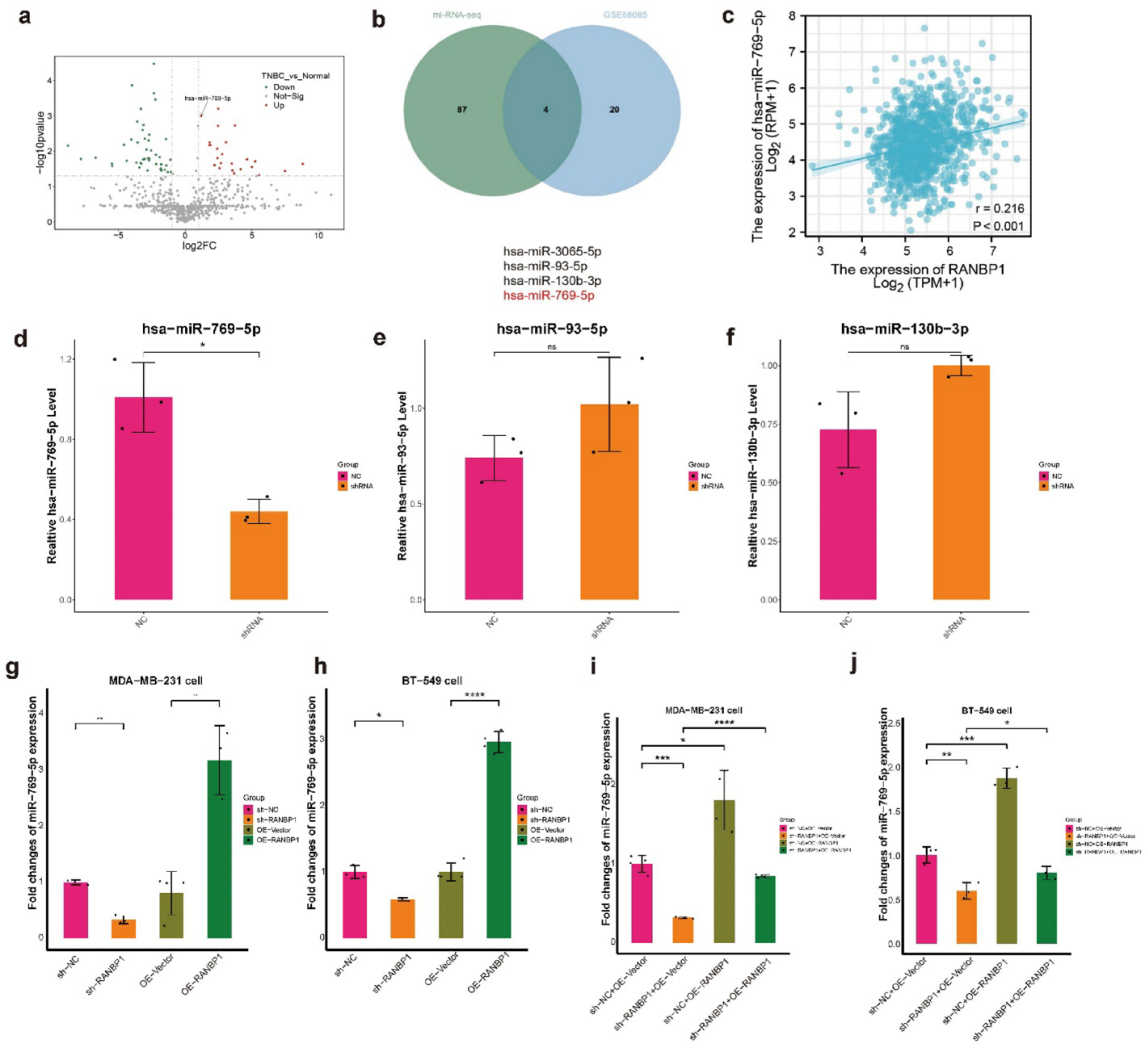
RANBP1 affects miRNA expression in breast cells. Volcano plots illustrate the differentially expressed miRNA regulated in GSE68085 (*p* < 0.05). **a** Green and red denote down-regulated and up-regulated miRNAs, respectively. **b** miRNA were selected following the analysis of miRNA-sequencing and GSE68085 data. **c** Correlations between RANBP1 and miR769-5p were analyzed in breast tissues. **d**-**f** qPCR results of miRNA in RANBP1 knockdown group and control group in MDA-MB-231 cells. miR769-5p expression levels were detected by RT-qPCR in RANBP1 knockdown and over-expressing in MDA-MB-231 cells (**g**) and BT-549 cells (**h**). Cells were grouped with sh-NC + OE-vector, sh-RANBP1 + OE-vector, sh-NC + OE-RANBP1 and sh-RANBP1 + OE-RANBP1. miR769-5p expression levels were detected were detected by RT-qPCR in MDA-MB-231 cells (**i**) and BT-549 cells (**j**)

Total RNA sequencing was conducted on RANBP1-knockdown and control TNBC cells, revealing 91 miRNAs that were significantly downregulated due to RANBP1 knockdown (BioProject ID: PRJNA1223670). Following this, an analysis was conducted on the tumor-associated miRNA dataset GSE68085 to explore the significance of these miRNAs in TNBC. The analysis demonstrated an increased expression of 24 miRNAs in tissues from TNBC (Fig. 5a, S7). The convergence of these two datasets revealed a persistent dysregulation of 4 specific miRNAs (Fig. 5b). Bioinformatics-based correlation analyses additionally showed miR-769-5p expression positively associates with RANBP1 levels (Fig. 5c, S5c-e). Quantitative PCR analysis revealed that only miR-769-5p was differentially expressed (Fig. 5d-f), leading to the selection of miR-769-5p for subsequent analyses. Experimentally, silencing RANBP1 markedly reduced miR-769-5p expression in BT-549 and MDA-MB-231 cells, whereas increased expression of RANBP1 significantly elevated miR-769-5p levels (Fig. 5g, h). Moreover, rescue assays demonstrated that co-transfection with sh-RANBP1 and OE-RANBP1 effectively restored miR-769-5p levels compared to transfection with sh-RANBP1 alone (Figs. 5i, j). Taken together, these data support the notion that miR-769-5p expression is positively regulated by RANBP1 within TNBC cells.

### RANBP1 promoted TNBC cell proliferation and metastasis through miR-769-5p

**Fig. 6:**
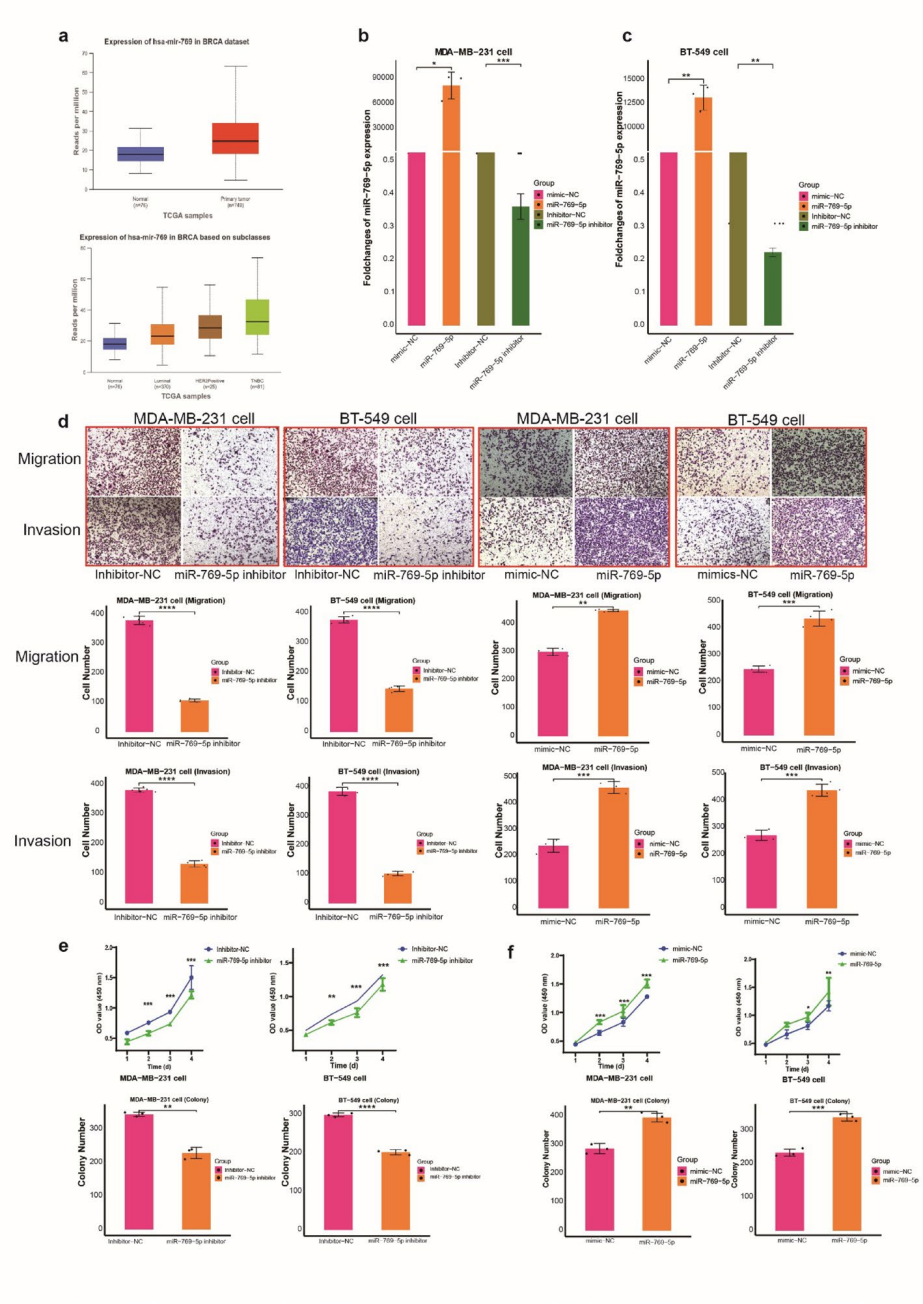
Effect of miR-769-5p on the proliferation and migration of the BC cells. **a** Higher miR-769-5p expression was detected in breast tissues from TCGA databa. miR-769-5p expression were detected in miR-769-5p mimic or miR-769-5p inhibitor in MDA-MB-231 cells (**b**) and BT549 cells (**c**) by qRT-PCR. Transwell assays were performed to detect the migration and invasion of miR-769-5p inhibitor or miR-769-5p mimic (**d**) in MDA-MB-231cells and BT-549 cells. Colony formation assay and CCK8 assay was applied to measure the MDA-MB-231cells (**e**) and BT-549 cells (**f**) proliferation in miR-769-5p mimic or miR-769-5p inhibitor

The examination of datasets available in public repositories revealed notably elevated expression levels of miR-769-5p within TNBC tumor samples (Fig. 6a). To further determine the biological role of miR-769-5p, mimics and inhibitors specific to this microRNA were introduced into MDA-MB-231 and BT-549 cells through transfection (Fig. 6b, c). The invasive and migratory abilities were significantly attenuated in cells following miR-769-5p inhibition; conversely, these abilities markedly increased upon miR-769-5p mimic treatment (Fig. 6d). Moreover, enhancing miR-769-5p expression boosted cell growth and colony-forming potential, whereas suppressing miR-769-5p led to opposite effects (Figs. 6e, f, S8). These combined findings strongly support the hypothesis that miR-769-5p facilitates proliferation and metastasis in TNBC cells.

### PRUNE2 expression negatively correlated with miR-769-5p levels in BC

**Fig. 7:**
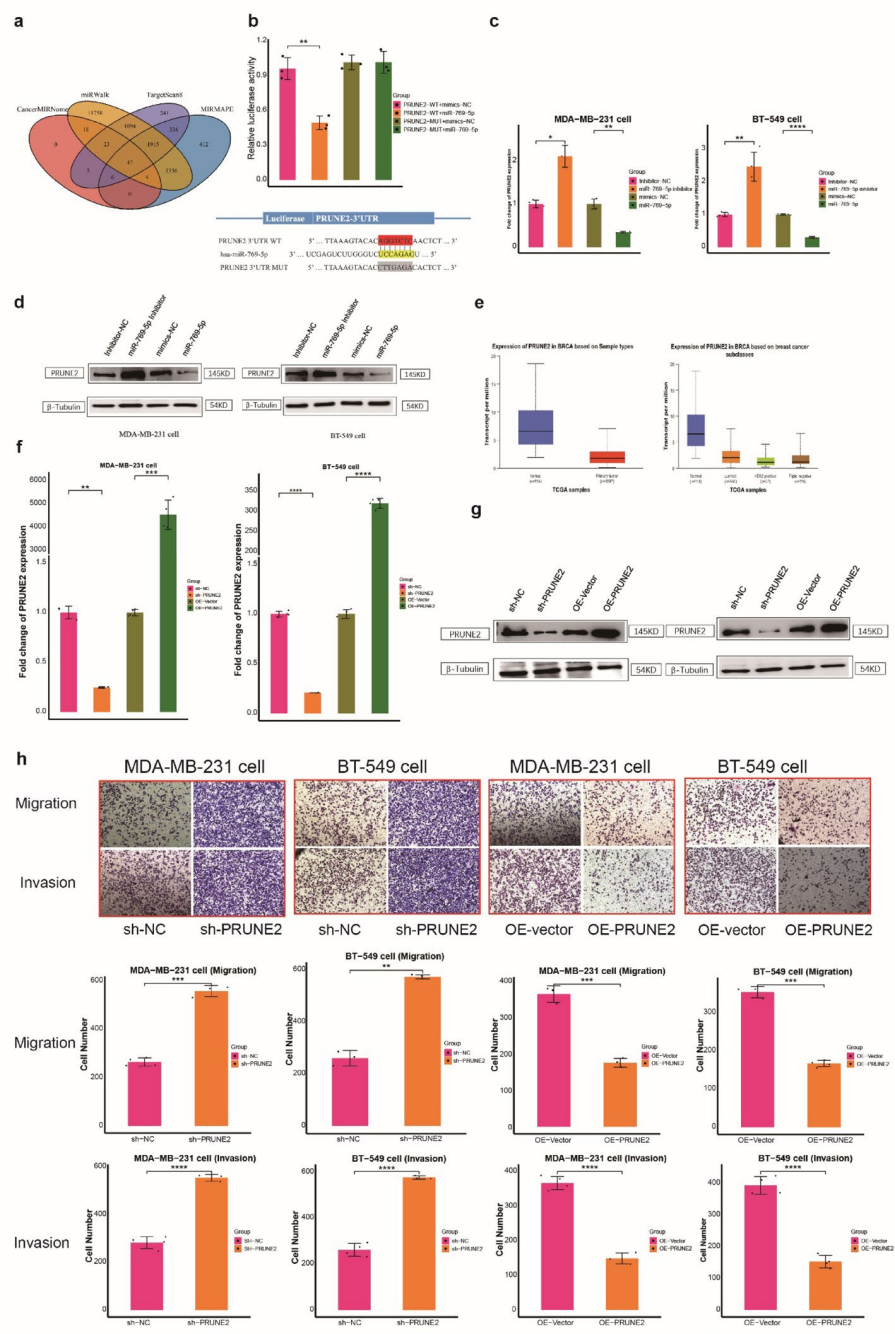
PRUNE2 is a direct target of miR-769-5p. **a** PRUNE2 targeted by miR-769-5p were estimated by cross-analysis with TargetScan, miRmap and miRwalk databases. **b** 293T was co-transfected with miR-769-5p mimics and Mut or WT PRUNE2 3’UTR LR vectors, and the activities of the LR were detected.The expression levels of PRUNE2 were assessed by RT-qPCR(**c**)and WB (**d**༉transfected with miR-769-5p inhibitor or mimic in MDA-MB-231cells and BT-549 cells. Lower PRUNE2 expression was detected from TCGA databa (**e**). Expression of PRUNE2 silencing or over-expression was detected in MDA-MB-231 and BT-549 cells by qRT-PCR(**f**) and by WB(**g**). Transwell assays were conducted to measure the migration and invasion capability in PRUNE2-knockdown or over-expression (**h**) in BT549 cells and MDA-MB-231 cells

Bioinformatics analysis using the TargetScan, CancerMIRNome, MIRMAPE, and miRWalk databases predicted a total of 47 target genes (Fig. 7a). The differential expression of these 47 target genes between tumors and controls was verified using the GEPIA database (http://gepia.cancer-pku.cn/). The results showed downregulation of HOXD3, PRUNE2, CD302, CCDC8, and KLF2 (tumor vs. control). Subsequent qPCR analysis of clinical samples revealed that only PRUNE2 showed a consistent and significant expression trend, making it a candidate gene potentially targeted by miR-769-5p (Fig. S5f). Furthermore, dual-luciferase reporter experiments demonstrated that luciferase activity driven by wild-type PRUNE2 3’UTR was markedly diminished following miR-769-5p overexpression in HEK293T cells, whereas the mutant PRUNE2 3’UTR construct showed no change in luciferase signal (Fig. 7b). In line with these observations, q-PCR and WB assays confirmed that introducing miR-769-5p mimics resulted in a substantial reduction of PRUNE2 protein and mRNA levels, while miR-769-5p inhibition produced the opposite pattern (Figs. 7c, d). These results collectively imply that PRUNE2 serves as a direct downstream target negatively regulated by miR-769-5p in TNBC.

**Fig. 8:**
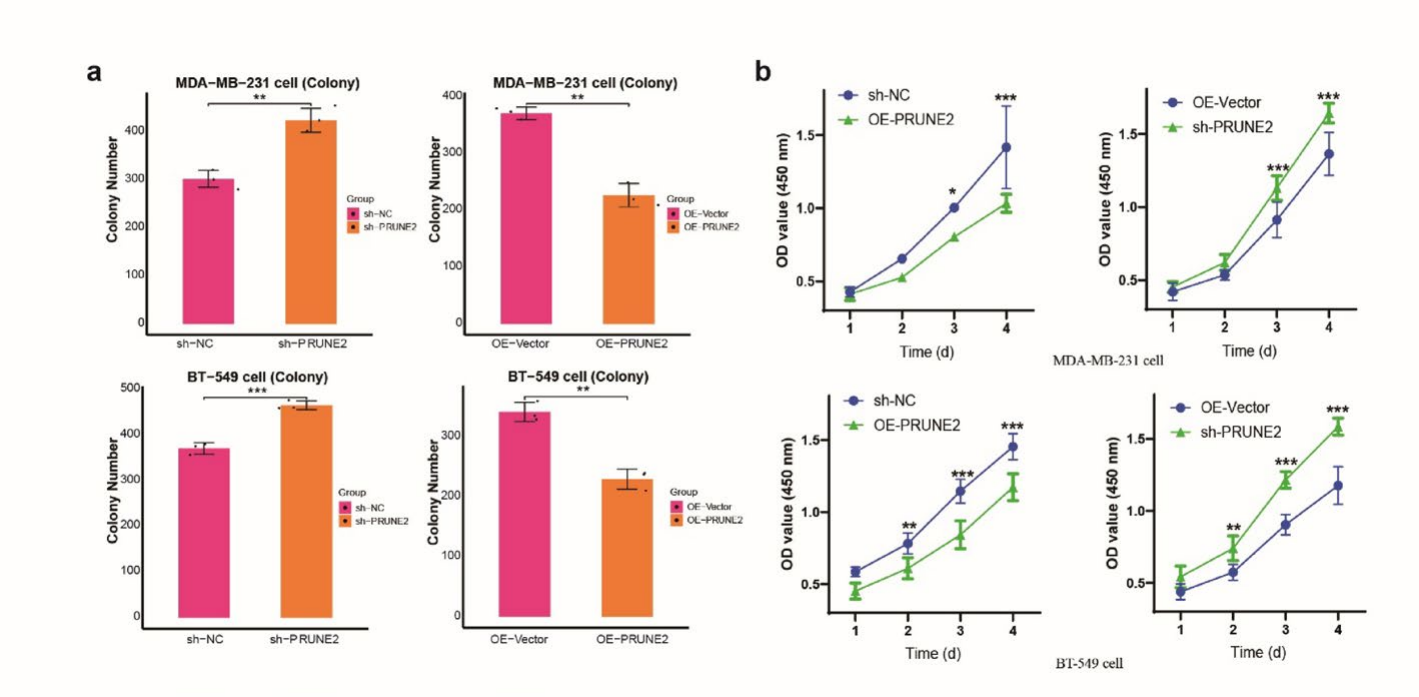
Colony formation were conducted in PRUNE2-knockdown (**a**) or over-expression (**b**) in BT549 cells and MDA-MB-231 cells

Tumor samples from the TCGA database had lower PRUNE2 expression than non-cancerous samples (Fig. 7e). Cells with PRUNE2 over-expression or knock out were constructed (Fig. 7f, g) and rates of cell proliferation and migration found to be lower for the former cell-type (Figs. 7h and 8a and b, S9 ). PRUNE2 may influence TNBC progression and is associated with the actions of miR-769-5p.

### RANBP1/miR-769-5p/PRUNE2 axis

**Fig. 9:**
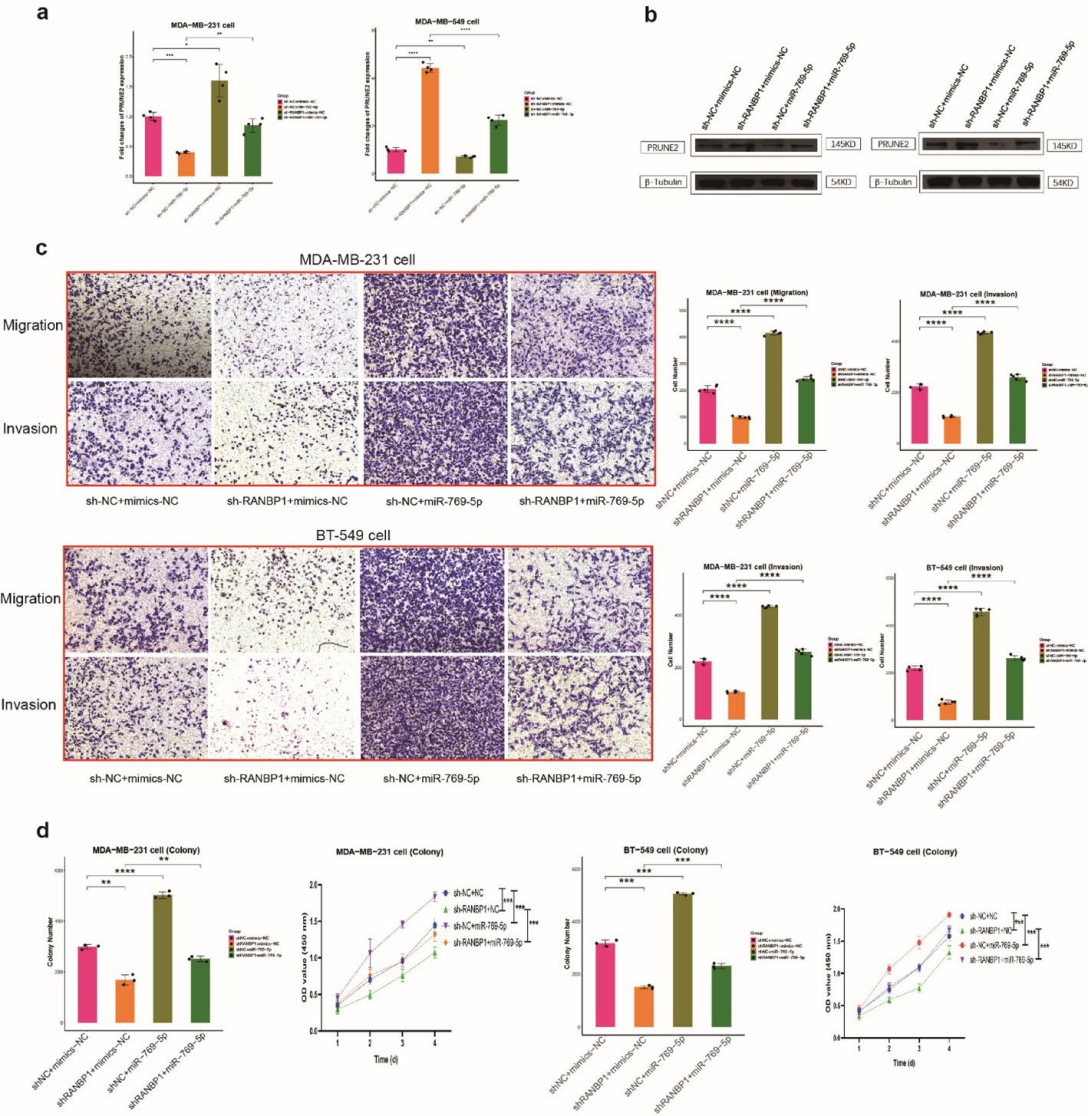
RANBP1 promotes proliferation, migration, and invasion in TNBC through the MiR-769-5p/PRUNE2 Axis The expression levels of PRUNE2 were assessed by RT-qPCR(**a**)and WB (**b**) in RANBP1 knocked down transfected with miR-769-5p mimic or mimics-NC in MDA-MB-231cells and BT-549 cells. Cells were grouped with sh-NC + NC, sh-RANBP1 + NC, sh-NC + miR-769-5p and sh-RANBP1 + miR-769-5p. **c** transwell assay colony formation and CCK8 (**d**) were detected in, respectively MDA-MB-231cells and BT-549 cells

Knock-down of RANBP1 in BT-549 and MDA-MB-231 cells resulted in significantly greater PRUNE2 expression which was reduced by miR-769-5p upregulation(Fig. 9a, b). In addition, miR-769-5p over-expression rescued the cell phenotype associated with sh-RANBP1 transfection, restoring rates of cell migration, invasion and colony formation in vitro (Fig. 9c, d, S10). The results are consistent with a regulatory effect of RANBP1 on PRUNE2 expression mediated via miR-769-5p.

### RANBP1 knockdown inhibited BC tumor growth in vivo

**Fig. 10:**
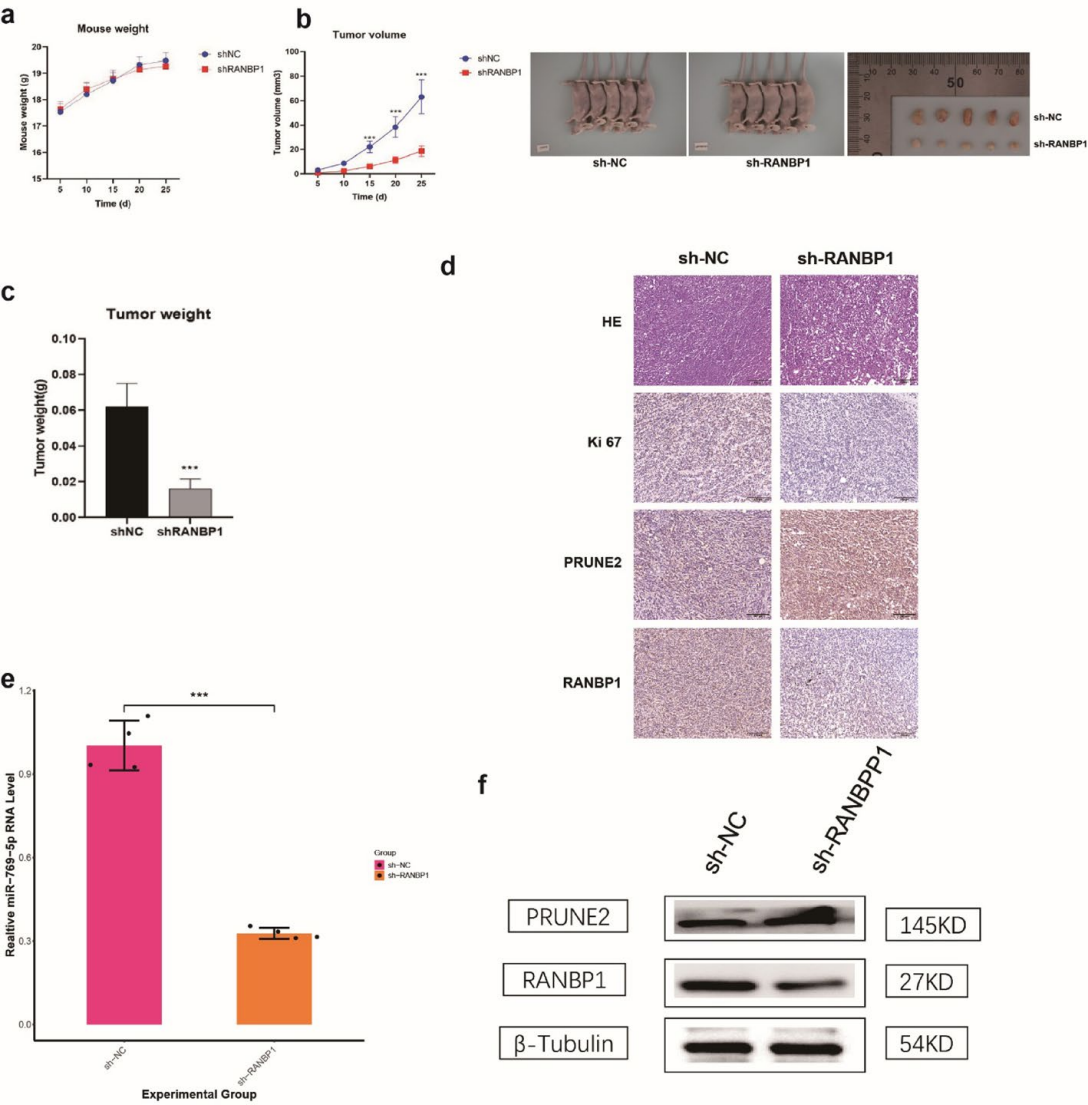
RANBP1 knockdown inhibits BC tumour growth in vivo. **a** Representative images of the tumors after subcutaneous injection with RANBP1-knockdown or NC in MDA-MB-231 cells. **b** Tumor volume and (**c**) weight was analyzed in RANBP1-knockdown/control group. **d** Representative images of IHC and H&E staining using antibodies against RANBP1, PRUNE2 and Ki-67. **e** qRT-PCR analysis of miR-769-5p expression in tumors. **f** RANBP1 and PRUNE2 were detected via WB

Mouse xenograft tumors were constructed by inoculation with the stable RANBP1 knockdown MDA-MB-231 cell line. No effect on mouse body weight was found as a result of RANBP1 knock out (Fig. 10a) but tumor volume and weight were lower than for control tumors (Fig. 10b, c). HE and IHC staining demonstrated that decreased RANBP1 expression corresponded with reduced Ki-67, a proliferation marker in tumor cells (Fig. 10d). Additionally, tissues from the sh-RANBP1 group exhibited diminished miR-769-5p expression (Fig. 10e) and concurrently elevated PRUNE2 levels (Fig. 10f). These results collectively support the hypothesis that RANBP1 modulates PRUNE2 through a miR-769-5p-dependent regulatory mechanism.

## Discussion

Patients exhibiting high RANBP1 expression were, on average, approximately 9.5 years older at diagnosis than those with low expression. Survival analysis indicated significantly lower 5-year OS rates for patients within the RANBP1-high subgroup (*P* = 0.0002). At study initiation, 50 of 56 patients from the RANBP1-low group remained alive compared to only 18 of 31 in the RANBP1-high group. Furthermore, most deaths in the high-expression cohort occurred within five years post-surgery, underscoring a clear relationship between elevated RANBP1 expression and poorer TNBC prognosis. Pathological assessment also identified significant histological staging differences between these two patient groups (*P* < 0.0001). Specifically, only 5 patients in the low-expression group were classified as stage III, whereas in the high-expression group, 13 patients reached stage III or higher, including one patient who presented with distant metastasis. Although pathological T and M stages did not differ statistically between groups (*P* = 0.1752, *P* = 0.3636), notable distinctions emerged in N staging. In the low-RANBP1 group, only 13 patients exhibited lymph node involvement (N1–N3), among whom 11 were classified above N2 stage (*P* = 0.0009). Collectively, these data indicate that enhanced RANBP1 expression may contribute to TNBC progression, correlating with adverse survival outcomes. Given the relatively limited sample size in this clinical cohort, larger-scale follow-up studies are warranted to further validate these findings.

TNBC may be classified into different subtypes [17] which are associated with different therapeutic targets and the high level of molecular heterogeneity renders TNBC unfavorable for targeted therapeutics. The Wnt, PTEN and PI3K/AKT pathways have all been implicated in TNBC growth, progression and metastasis [18] miRNAs have considerable therapeutic potential and have been identified as oncogene suppressors, tumor suppressors and signaling molecules [19–21].However, roles of RANBP1 and underlying molecular mechanisms remain unclear although this protein may have therapeutic potential via its impact on miRNA synthesis.

RANBP1 has been reported to regulate pre-miRNA nuclear export, which may subsequently influence epigenomic modifications [22]. Our previous research demonstrated that elevated RANBP1 expression independently predicted poor colorectal cancer prognosis, promoted tumor cell proliferation and invasion, and inhibited apoptosis [23]. Here, we provide the first evidence indicating a substantial and selective increase of RANBP1 expression in BC. Importantly, BC tissues with elevated RANBP1 levels exhibited increased malignancy and worse clinical outcomes. Consistent with these clinical findings, our experimental analyses revealed significantly elevated expression of RANBP1 in TNBC tissues. Functional assays demonstrated that RANBP1 knockdown markedly inhibited tumor cell proliferation and migratory capacity. Collectively, these results support the potential utility of RANBP1 as both a prognostic biomarker and therapeutic target in BC management.

Through integrated miRNA sequencing, analysis of TCGA and GEO databases, and correlation studies, we identified miR-769-5p as a critical downstream effector of RANBP1’s oncogenic function in BC cells. Our experiments revealed that silencing RANBP1 significantly reduced miR-769-5p expression, suggesting miR-769-5p as a direct regulatory target of RANBP1 in BC. Previous studies have indicated miR-769-5p’s oncogenic role in various cancer types; notably, miR-769-5p promoted tumor progression in 62 gastric cancer patients by suppressing YY-1 binding protein expression [24]. Moreover, this microRNA has been implicated in tumorigenesis and metastatic behaviors in lung cancer, prostate cancer, and osteosarcoma [25–27]. Consistently, in alignment with our study findings, miR-769-5p exhibited significant up-regulation in TNBC tissues. Results from CCK-8 assays, plate cloning, invasiveness and migration capability collectively underscored its pro-oncogenic characteristics.

Additionally, PRUNE2 was confirmed as an immediate downstream target modulated by the RANBP1/miR-769-5p signaling pathway in TNBC through integrative bioinformatics analyses and experimental validations. Animal-based experiments further demonstrated that silencing RANBP1 expression, accompanied by decreased miR-769-5p levels, markedly elevated PRUNE2 expression in vivo. PRUNE2 belongs to the PRUNE family of genes, which are implicated in neuronal synapse regulation, apoptotic processes, and malignant transformation [28, 29]. Furthermore, previous research has reported PRUNE2 as a tumor suppressor that is negatively modulated by PCA3 in prostate cancer [30]. Recent studies have also suggested PRUNE2 as a predictive and prognostic biomarker in ovarian, thyroid, colorectal cancers, and choriocarcinoma [31–33]. PRUNE2 may also regulate the JAK-STAT pathway, affecting cell motility in oral cancer [33]. However, further studies are needed to identify PRUNE2 function in breast tumor pathogenesis. PRUNE2 expression was down-regulated by the bioinformatics results. PRUNE2 reduced BC cell proliferation, migration and invasion.

Although our study establishes the RANBP1/miR-769-5p/PRUNE2 axis as a central finding, the precise mechanisms by which RANBP1 regulates miR-769-5p remain to be clarified. Evidence from prior studies suggests at least two plausible pathways. First, RANBP1 functions as an essential cofactor in the RanGTPase system, maintaining the RanGTP/RanGDP gradient that drives nucleo-cytoplasmic transport. In this context, RANBP1 may influence the export of precursor miRNAs (pre-miRNAs) by facilitating Exportin-5–mediated nuclear export, thereby enhancing the maturation and stability of specific miRNAs [34, 35]. Indeed, disruption of Ran-dependent nuclear export has been shown to cause nuclear accumulation of pre-miRNAs and reduced levels of mature miRNAs, a process that can be rescued by enforced Exportin-5 expression. Second, RANBP1 has been implicated in a positive feedback loop with the Hippo/YAP pathway, where YAP nuclear localization is promoted by RANBP1-dependent signaling, and in turn, YAP/TEAD complexes transcriptionally activate RANBP1 [34]. Given that Hippo/YAP signaling has emerged as a regulator of Microprocessor activity and pri-miRNA processing, this axis may indirectly modulate miR-769-5p transcription or co-transcriptional processing [34]. Furthermore, RANBP1’s broader roles in RNA metabolism and nuclear transport raise the possibility that RNA-binding proteins or cytoskeletal feedback mechanisms contribute to miRNA biogenesis regulation [35, 36]. Taken together, these observations support a working model in which RANBP1 promotes miR-769-5p expression both by facilitating its nuclear export and by engaging signaling cascades that regulate miRNA transcription and processing. Future studies, such as nuclear/cytoplasmic fractionation combined with pre-/mature miRNA quantification, XPO5 rescue experiments, and ChIP-qPCR of YAP/TEAD binding near the MIR769 locus, will be valuable to validate these hypotheses and to refine our understanding of how RANBP1 shapes the functional output of the RANBP1/miR-769-5p/PRUNE2 axis in tumorigenesis.

## Conclusions

In summary, RANBP1 critically modulates the tumor immune microenvironment in TNBC, and its overexpression strongly correlates with adverse clinical outcomes. Mechanistically, RANBP1 enhances miR-769-5p levels, promoting tumor growth and invasion by downregulating PRUNE2. This signaling pathway—RANBP1/miR-769-5p/PRUNE2—represents a promising potential therapeutic target in TNBC treatment strategies.

## Supplementary Information

Below is the link to the electronic supplementary material.

Supplementary Material 1.

Supplementary Material 2.

Supplementary Material 3.

Supplementary Material 4.

Supplementary Material 5.

Supplementary Material 6.

## Data Availability

The initial dataset has been submitted to the NCBI repository PRJNA1223670 (https://www.ncbi.nlm.nih.gov/sra/PRJNA1223670).

## Abbreviations

BC: Breast cancer
TNBC: Triple-negative breast cancer
RANBP1: Ran-binding protein 1
PRUNE2: Prune homolog 2
DMEM: Dulbecco’s modified essential medium
FBS: Fetal bovine serum
PVDF: Polyvinylidene difluoride
