# Supplementary figures and images for: RANBP1 promotes immune evasion in triple-negative breast cancer by suppressing T cell infiltration via the miR-769-5p/PRUNE2 axis
Source: Discov Oncol. 2025 Nov 4;16:2026. doi: 10.1007/s12672-025-03872-7 (PMC12586255; doi:10.1007/s12672-025-03872-7)

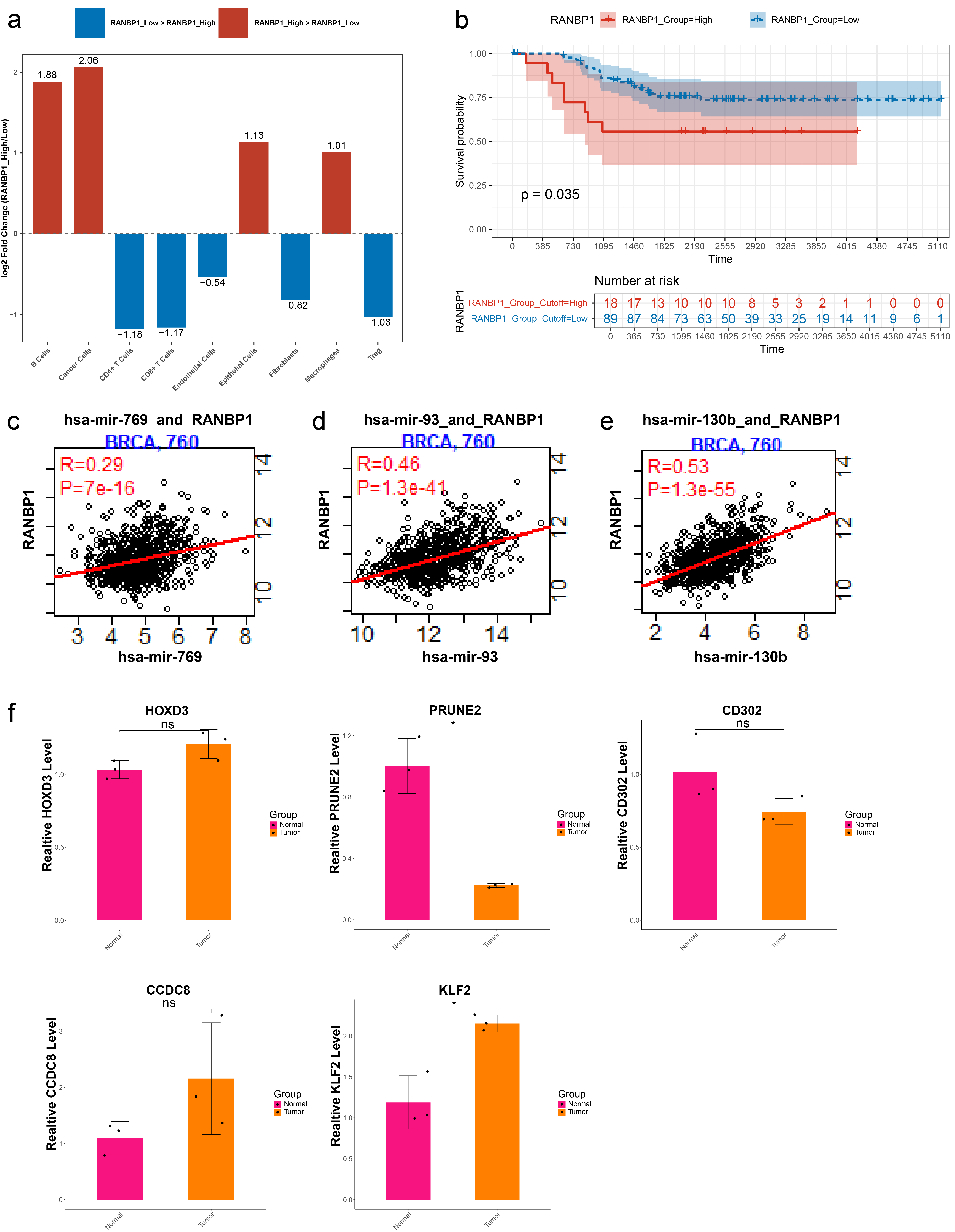

Supplement: Supplementary file 4 — Supplementary Material 4. [file 12672_2025_3872_MOESM4_ESM.jpg]

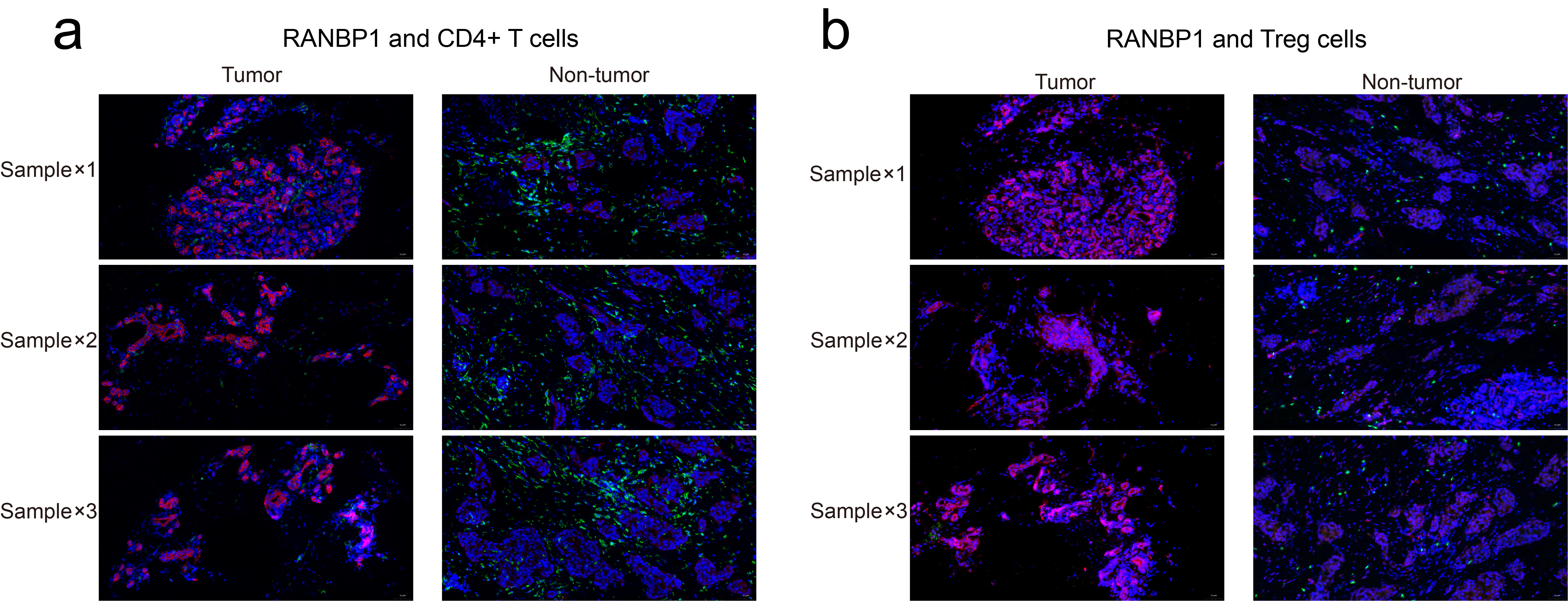

Supplement: Supplementary file 5 — Supplementary Material 5. [file 12672_2025_3872_MOESM5_ESM.jpg]

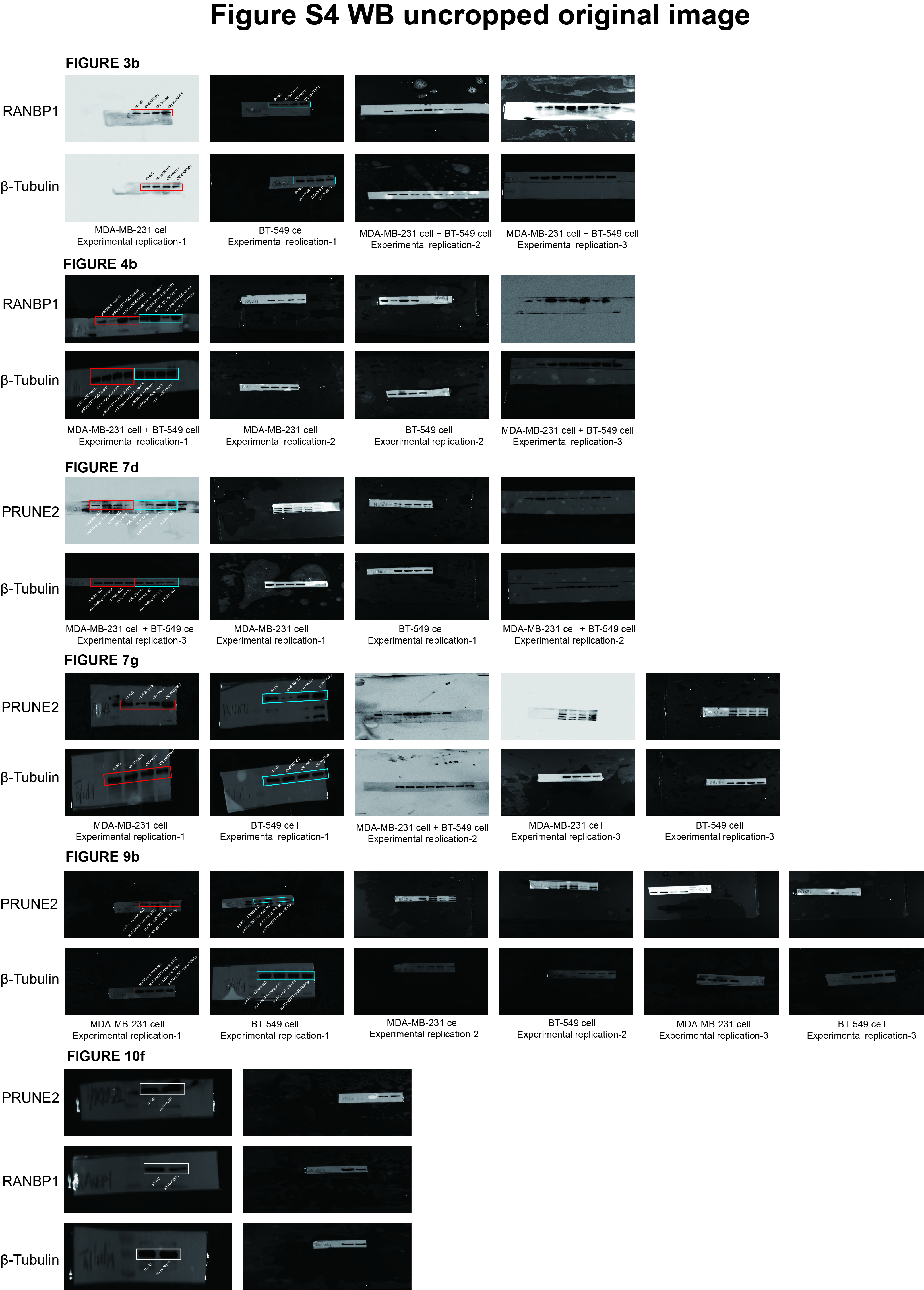

Supplement: Supplementary file 6 — Supplementary Material 6. [file 12672_2025_3872_MOESM6_ESM.jpg]
